# Physics-informed machine learning for automatic model reduction in chemical reaction networks

**DOI:** 10.1038/s41598-025-92680-8

**Published:** 2025-03-07

**Authors:** Joseph Pateras, Colin Zhang, Shriya Majumdar, Ayush Pal, Preetam Ghosh

**Affiliations:** 1 Department of Computer Science, Virginia Commonwealth University, Richmond, Virginia 23284 USA; 2 Department of Electrical Engineering and Computer Sciences, University of California, Berkeley, Berkeley, CA 94720 USA; 3 Department of Computer Science, University of Texas at Austin, Austin, TX 78712 USA; 4 Mills E. Godwin High School, Richmond, Virginia 23238 USA

**Keywords:** Biophysics, Systems biology, Biochemical networks, Computer modelling, Computer science, Computer science, Scientific data

## Abstract

Physics-informed machine learning bridges the gap between the high fidelity of mechanistic models and the adaptive insights of artificial intelligence. In chemical reaction network modeling, this synergy proves valuable, addressing the high computational costs of detailed mechanistic models while leveraging the predictive power of machine learning. This study applies this fusion to the biomedical challenge of A$\beta$ fibril aggregation, a key factor in Alzheimer’s disease. Central to the research is the introduction of an automatic reaction order model reduction framework, designed to optimize reduced-order kinetic models. This framework represents a shift in model construction, automatically determining the appropriate level of detail for reaction network modeling. The proposed approach significantly improves simulation efficiency and accuracy, particularly in systems like A$\beta$ aggregation, where precise modeling of nucleation and growth kinetics can reveal potential therapeutic targets. Additionally, the automatic model reduction technique has the potential to generalize to other network models. The methodology offers a scalable and adaptable tool for applications beyond biomedical research. Its ability to dynamically adjust model complexity based on system-specific needs ensures that models remain both computationally feasible and scientifically relevant, accommodating new data and evolving understandings of complex phenomena.

## Introduction

Deriving models for complex processes is a hallmark of modern science. Cleverly devised modeling affords viability to the computational analysis of many complex physical, chemical, biological, and geological systems. As system complexity grows, the dimensionality of our models must also increase. In the context of Alzheimer’s disease (AD), particularly the pathological aggregation of Amyloid-$\beta$ (A$\beta$) peptides, this study leverages advanced computational biology and machine learning methods to refine our understanding and modeling of A$\beta$ fibril formation. The problem presented here belongs to a large class of multiphysics problems, which might be computationally difficult due to scale, underpinning dynamics, or observational difficulty.

The aggregation of A$\beta$ peptides, a central event in AD pathology, is characterized by the accumulation of insoluble fibrils detrimental to neuronal cells. This process poses a significant challenge due to the intricate biochemical pathways involved and the difficulty in observing them in vivo. Traditional modeling approaches, while valuable, often fall short in capturing the full dynamics of A$\beta$ aggregation. This shortfall necessitates the development of novel strategies for model refinement and analysis. To address this, we employ physics-informed machine learning paradigms aimed at automatic model order reduction. Our focus is on accurately representing primary and secondary nucleation states-key processes in fibril aggregation involving the formation of nucleation centers and the generation of new nucleation sites on existing fibrils. By integrating accelerated and reliable parameter estimation techniques, this research aims to elucidate the nuanced dynamics of reduced-order A$\beta$ aggregation models, contributing to a deeper understanding of AD mechanisms and the exploration of potential therapeutic interventions.

In the evolving landscape of computational biology and machine learning, the fusion of data-driven approaches with traditional scientific modeling offers new opportunities for understanding complex biological processes. One of the most pressing challenges in modern medicine is unraveling Alzheimer’s disease mechanisms, particularly the role of A$\beta$ fibril aggregation. This process, central to AD pathology, involves the accumulation and deposition of A$\beta$ peptides into insoluble fibrils, which are toxic to neuronal cells. Despite extensive research, the precise dynamics and regulatory mechanisms of A$\beta$ aggregation remain only partially understood, due to the complexity of the underlying biochemical pathways and the limitations of conventional modeling techniques^1,2^. Although most proteins typically exist in soluble, monomeric forms or well-defined complexes, some functional amyloid states are insoluble and pathological^3^.

Ordinary differential equation modeling serves as a powerful tool in the study of amyloid-$\beta$ fibril aggregation: a critical process implicated in the pathogenesis of Alzheimer’s disease. These models can elucidate key parameters such as nucleation rates, growth kinetics, and the influence of various environmental factors, ultimately contributing to a deeper understanding of the disease and the potential development of therapeutic interventions^4^. State-of-the-art analytical frameworks, such as Amylofit^5^, utilize comprehensive rate equations to model the dynamics of filamentous assembly. These frameworks underscore three pivotal mechanisms influencing aggregation kinetics. First, primary nucleation is identified, a process solely influenced by the abundance of free monomers. An instance of this mechanism is the formation of nucleation centers in supersaturated solutions. Next, secondary nucleation is discussed, a phenomenon that is governed by both the availability of free monomers and the presence of pre-formed aggregate structures. An example of secondary nucleation is the formation of new nucleation sites on the surfaces of existing fibrils. Finally, the concept of fragmentation is introduced, a process that is independent of monomer concentration and relies solely on the existing aggregated mass. However, the relative importance of each process, and their modeling applicability are hyper-specific and depend upon the availability, veracity, and granularity of available data. As medical imaging, and the general observability of A$\beta$ proteins is enabled further, the tasks of parsing new data and prescribing appropriate models must maintain pace. This work, presented with data on transient concentrations of A$\beta$, proposes a framework by which primary and secondary nucleation distinctions can be informatively drawn.

The challenge of accurately modeling complex biochemical systems-such as the aggregation of A$\beta$ peptides into amyloid fibrils, a hallmark of Alzheimer’s disease, stems from their inherent multiphysics nature. These systems are characterized by intricate interactions across different physical, chemical, and biological domains. Traditional modeling techniques, while powerful, often struggle to capture the full spectrum of dynamics at play due to computational limitations and the difficulty of incorporating all relevant phenomena into a single model^6^.

Thus, we propose a physics-informed neural network (PINN) model for amyloid-$\beta$ parametric studies. This PINN architecture alleviates concerns in complex modeling through physics-informed biases, which allow learning paradigms to intuit relationships in incomplete, or noisy datasets, while increasing the speed and veracity of machine learning models^7,8^. Physics-informed machine learning emerges as a cutting-edge solution to the limitations faced by conventional modeling approaches^9,11^, offering a novel pathway to model complex systems like A$\beta$ aggregation with unprecedented accuracy and efficiency where hidden physics in complex multiphysics problems can be intuited by learning algorithms^12,13^.

In the context of A$\beta$ aggregation, PINNs represent a significant leap forward. One of the most compelling advantages of PINNs is their ability to overcome the computational and data-related challenges that hamper traditional modeling efforts. Ultimately, the integration of physics-informed machine learning into the study of A$\beta$ aggregation aims to catalyze a deeper understanding of Alzheimer’s Disease pathogenesis, promising to accelerate the development of effective treatments^14^.

By incorporating domain knowledge of chemical kinetics and reaction dynamics directly into the learning algorithms, we aim to develop a model that not only predicts the behavior of A$\beta$ aggregation with high accuracy but also adheres to the well-studied biophysical principles governing these processes. This approach enables the exploration of the aggregation phenomenon beyond the constraints of traditional computational models, offering insights into the kinetics of nucleation, growth, and the formation of toxic fibrils under varying conditions.

## Methods

### Reduced order chemical kinetic model

The uncontrolled aggregation of toxic amyloid fibrils is a condition underpinning many serious health conditions. An example of such a protein, which reacts with environmental catalysts in our brains, is called Amyloid-$\beta$ (A$\beta$). Modeling the formation of potentially toxic amyloid plaques is important^15^ as direct observation of this process is prohibitively invasive. In the year 1906, it was only during an autopsy when Dr. Alois Alzheimer noted the presence of these plaques in the brain of a patient exhibiting dementia like symptoms now synonymous with Alzheimer’s Disease.

**Fig. 1:**
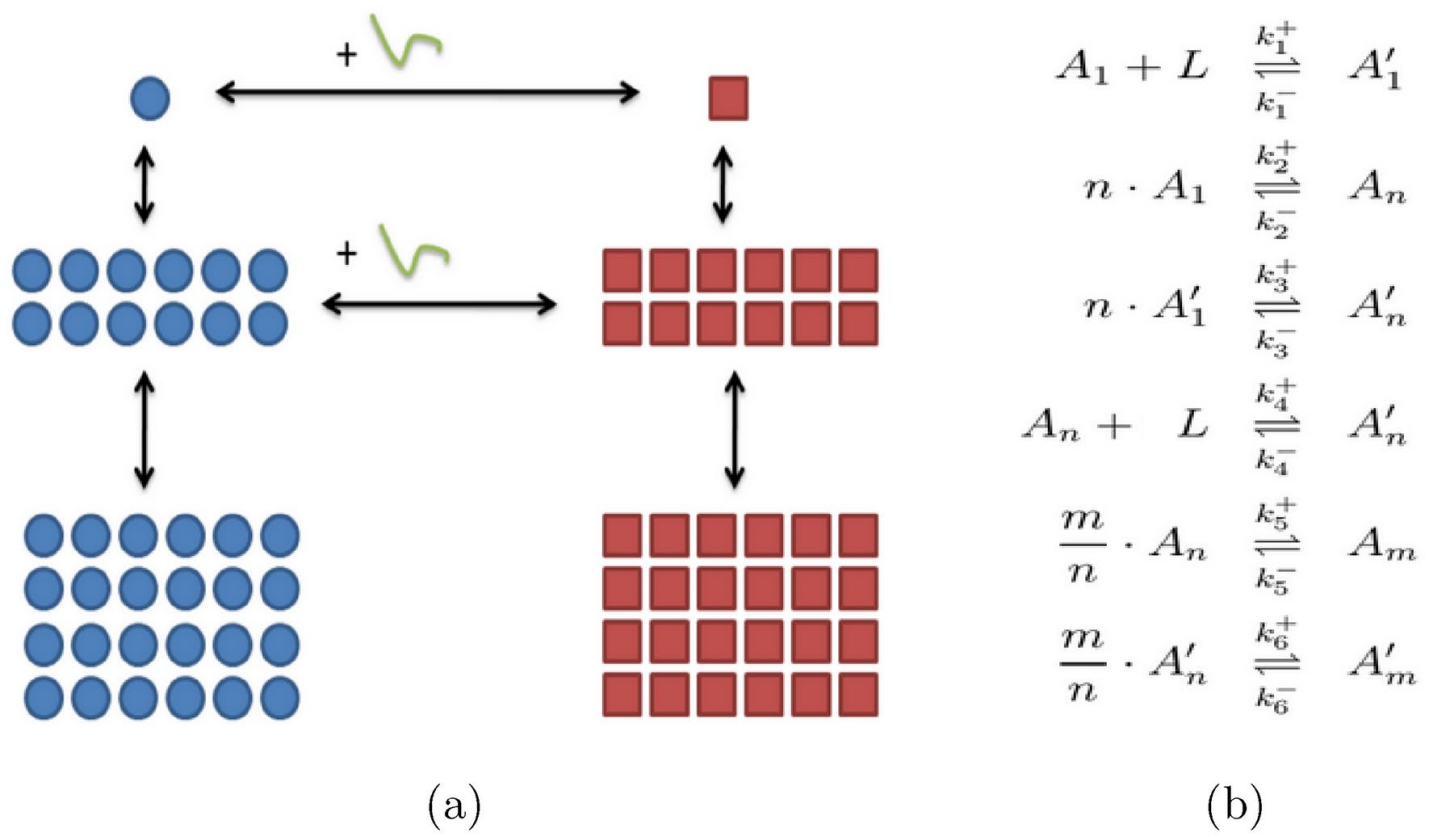
(**a**) A reduced order aggregation model for A$\beta$ amyloid and (**b**) the chemical reactions corresponding to the link between each aggregation step or pathway mutation.

The model presented in Fig. 1 is a reduced order representation of A$\beta$ aggregation. Reduced order representations are studied thoroughly to draw conclusions about competing aggregation processes^16,17^. Figure 1 gives the 3-species On-pathway model used in this study. Amyloid-$\beta$ aggregation is considered to form through polymerization reactions in the monomeric chemical species. The model aggregates are represented as three discrete chemical species. Reactions between species are represented by elementary rate parameters $\alpha$ and $\beta$, with forward and backward reaction rates respectively. Off-pathway intermediates are considered in a separate analysis.

Both healthy and toxic pathways culminate in a post-nucleation size oligomer of size *m* ($A_m$ and $A'_m$ respectively) with intermediate species size *n*. The reactions in Fig. 1b describe the interactions between the differently-sized aggregates of A$\beta$. Each double ended arrow in Fig. 1 describes a reversible aggregation reaction, or a switching between toxic and healthy pathways. It is observed that fully realized fibrils no longer mutate between healthy or toxic. Here *L* denotes the presence of environmental catalyst which caused the mis-formation of mature plaques along the Off-pathway.

Each arrow in our network abstraction corresponds to a coalescing or dissolving aggregation reaction. Employing the law of mass action we can convert the list of chemical reactions into a system of differential equations describing the concentration of each Amyloid-$\beta$ species.

The entire amyloid aggregation system is complex and a full-scale analysis is difficult to imagine. The aggregation of A$\beta$ into amyloid fibrils involves a series of biochemical reactions, including nucleation, elongation, and fibril branching. The full-scale simulation of every individual reaction and molecular interaction is computationally infeasible due to the sheer number of molecules and the stochastic nature of their interactions. Therefore, reduced order models are essential for understanding the key processes that drive A$\beta$ aggregation. These models abstract the aggregation process into a series of rate equations that describe the change in concentration of different species over time, focusing on critical transitions such as nucleation and fibril elongation.

Reduced order models are derived based on the assumption that certain steps in the aggregation process are rate-limiting, meaning they significantly affect the overall rate of aggregation. For example, the formation of a stable nucleus (the nucleation process) is often considered a critical step in the aggregation pathway. By identifying and modeling these key steps, researchers can gain insights into the mechanisms of aggregation and identify potential therapeutic targets to prevent or disrupt the formation of toxic amyloid fibrils. The importance of the mass-action framework for constructing the reduced order representation of the aggregation complex is given in Appendix A, where the equations governing the On- and Off-pathway toward aggregation are derived; and in Section “Automatic reaction order reduction” where the eight models studied here are explored in detail. Critically, the adaptability of the mass-action, reduced order model toward catalyzed Off-pathway aggregation make it a valuable tool in studying neurodegeneration linked to AD.

### PINN for parameter estimation

Machine learning approaches are widely used due to their ability to mitigate costs associated with large-scale models. In multiphysics systems, machine learning can explore high-dimensional feature spaces, with deep architectures offering creative ways to extract features from multi-fidelity data. Traditional data-driven methods, such as neural networks, rely solely on input–output relationships, which can falter when datasets are limited, noisy, or subject to physical constraints.

When empirical data is scarce or simulations are computationally expensive, physics-informed machine learning (PIML) combines the computational advantages of machine learning with systemic information to enhance generalization. Karniadakis et al.^9^ outline three principles of PIML: observational biases, inductive biases, and learning biases. The proposed PINN model for chemical reaction networks leverages learning biases informed by governing equations and inductive biases derived from aggregation kinetics knowledge, as categorized by the drivers described in Pateras et al.^10^.

By incorporating governing equations as constraints during training, PIML can estimate outcomes and time-dependent effects efficiently. This method has shown promise across domains like fluid dynamics, heat transfer, and materials science. For modeling systems like A$\beta$ aggregation, where data is sparse and biochemical processes are complex, PINNs provide a computationally efficient alternative to traditional simulations. This fusion of data-driven learning and physics-based constraints ensures parameter estimates are consistent with both data and physical laws.

In the context of A$\beta$ aggregation, PINNs can predict intermediate species concentrations over time, offering insights into nucleation, growth, and fibril formation. By integrating sparse data with differential equations governing aggregation, PINNs help uncover the kinetics of amyloid formation while reducing computational costs.

**Fig. 2:**
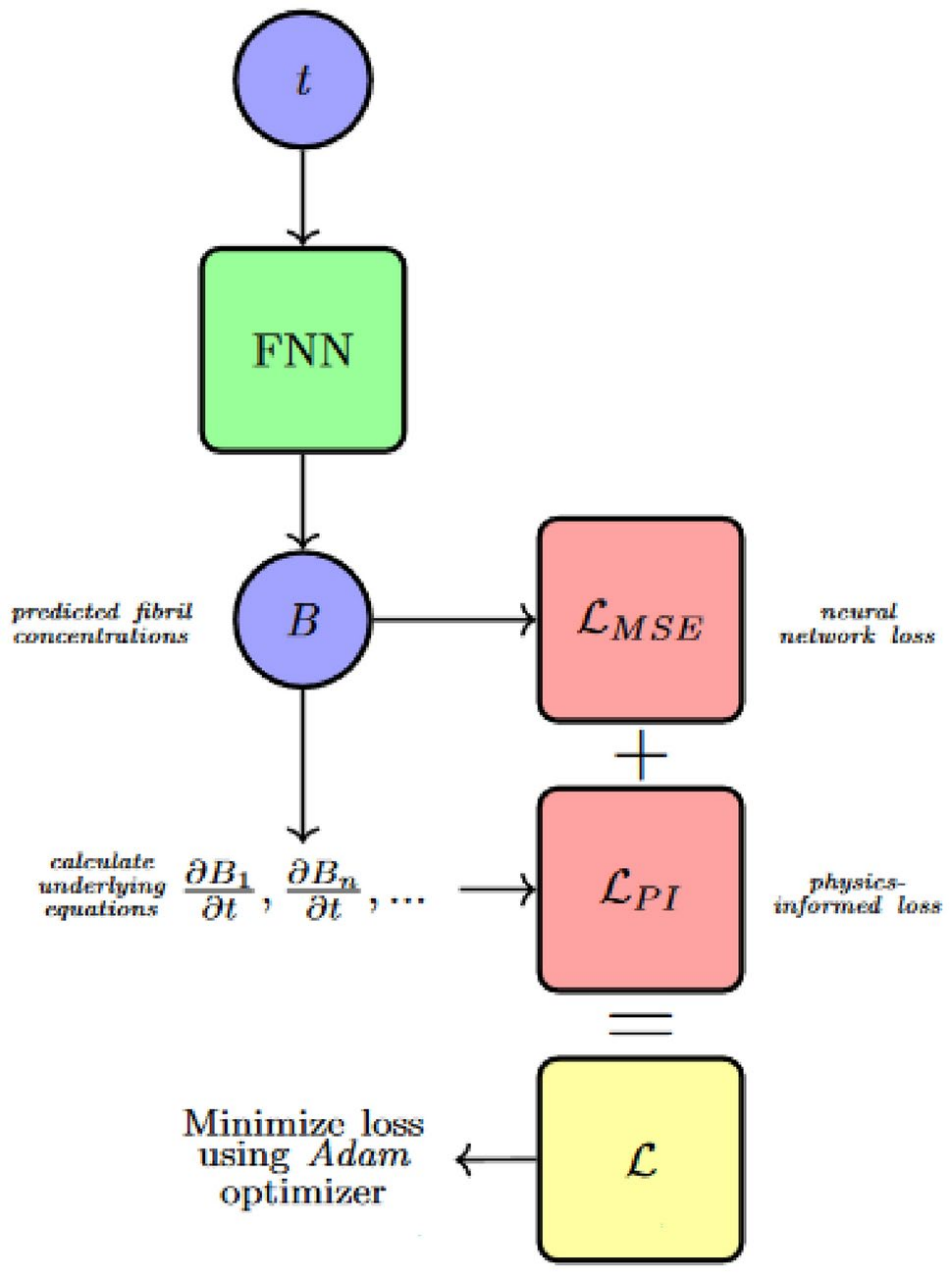
A diagram of physics-informed learning bias in the informed neural network routine. Observed aggregation is used to generate loss from data and from governing equations.

Figure 2 gives a schematic of the physics-informed learning bias process, which enables parameter estimation. Here the physics-informed loss is jointly optimized with neural network loss. Equations (1)–(2) describe the different computations performed. Physics-informed loss, $\mathcal {L}_{PI}$, forces the model to adhere to governing physical principles, while standard neural network loss, $\mathcal {L}_{MSE}$, directly learns correlations in training data.

By embedding the governing equations of A$\beta$ aggregation directly into the loss function of the neural network, PINNs can predict the dynamics of amyloid formation under various conditions. This method ensures that the predictions respect conservation laws, reaction kinetics, and other biophysical constraints, enhancing the model’s reliability and interpretability. The physics-informed loss component $\mathcal {L}_{PI}$ penalizes deviations from the physical model, guiding the learning process to conform to the biophysical reality of amyloid fibril formation. For *n*-many observations, $\hat{Y}_t$ and ${Y}_t$ denote neural network predicted concentrations and observed fibril concentrations respectively. *F* encodes the governing differential equations. Thus, forcing the loss to bias toward solutions which satisfy the underlying dynamics. While Eq. (2) directly compares predictions to training data. Thus, the total loss $\mathcal {L}$ is defined as $\mathcal {L} = \mathcal {L}_{MSE} + \mathcal {L}_{PI}$. Where:

1 $$\begin{aligned} & \mathcal {L}_{PI} = \frac{1}{n}\sum _{t=1}^{n}| F(Y_t) - F(\hat{Y}_t) | \end{aligned}$$

2 $$\begin{aligned} & \mathcal {L}_{MSE} = \frac{1}{n}\sum _{t=1}^{n}(Y_t-\hat{Y}_t)^2 \end{aligned}$$

Incorporating PINNs into the study of A$\beta$ aggregation offers a novel approach to understanding and predicting the dynamics of amyloid fibril formation. This methodology not only provides a pathway to estimate unknown parameters with high accuracy but also opens new avenues for exploring the mechanistic underpinnings of neurodegenerative diseases. This approach enhances reliability and interpretability by ensuring that predictions align with both observed data and biophysical reality.

### Automatic reaction order reduction

We formalize the proposed automatic model reduction framework by answering the following question: Given observations of amyloid concentrations with various molecular weights, what is the optimal choice of modeling scale? Namely, how should primary and secondary nucleation species be subdivided producing a reliably balanced model capturing both complexity and model granularity?

Given the influence of nucleation in rate limiting the aggregation process, it is recognized the model must differentiate between pre-nucleation and post-nucleation events to capture the essential physics of A$\beta$ aggregation. The process of nucleation is a cornerstone in the aggregation of A$\beta$, serving as a rate-limiting step that significantly influences the overall dynamics of aggregation. The distinction between primary-nucleation and secondary-nucleation events is not merely a technical detail but a fundamental aspect that affects how models predict the formation and growth of amyloid fibrils. Pre-nucleation interactions lead to the formation of oligomers, which then transition through a nucleation event to form the seeds for fibril elongation. Differentiating between these stages is crucial for understanding how initial conditions and environmental factors influence the aggregation pathway and the formation of toxic versus non-toxic species. The accurate modeling of these stages is critical for understanding the initiation and progression of amyloid diseases, making the choice of modeling scale a question of paramount importance. Thus by harnessing domain specific knowledge about nucleation and utilizing learning machines which facilitate the fusion of physical priors and optimization algorithms, we can answer the question presented above with domain-specific intuition and with learning machine optimality.

The 3-species on-pathway model has been studied for certain datasets. Reduced order models for ordered aggregation of A$\beta$ are constructed based on assumptions that identify the rate-limiting steps both before and after nucleation, which are critical in the formation and aggregation process of amyloid fibrils. These models simplify the complex mechanisms of amyloid beta aggregation by focusing on key transitional states, such as the formation of oligomers and their assembly into fibrils, underpinning the aggregation pathway. The efficacy of these models in extrapolating off-pathway aggregation and amyloidosis lies in their ability to predict the behavior of A$\beta$ under various conditions. By focusing on rate-limiting steps, these models help in understanding how deviations from the primary pathway lead to disease-relevant off-pathway aggregates, offering a valuable tool for the development of strategies to combat amyloid-related diseases.

**Fig. 3:**
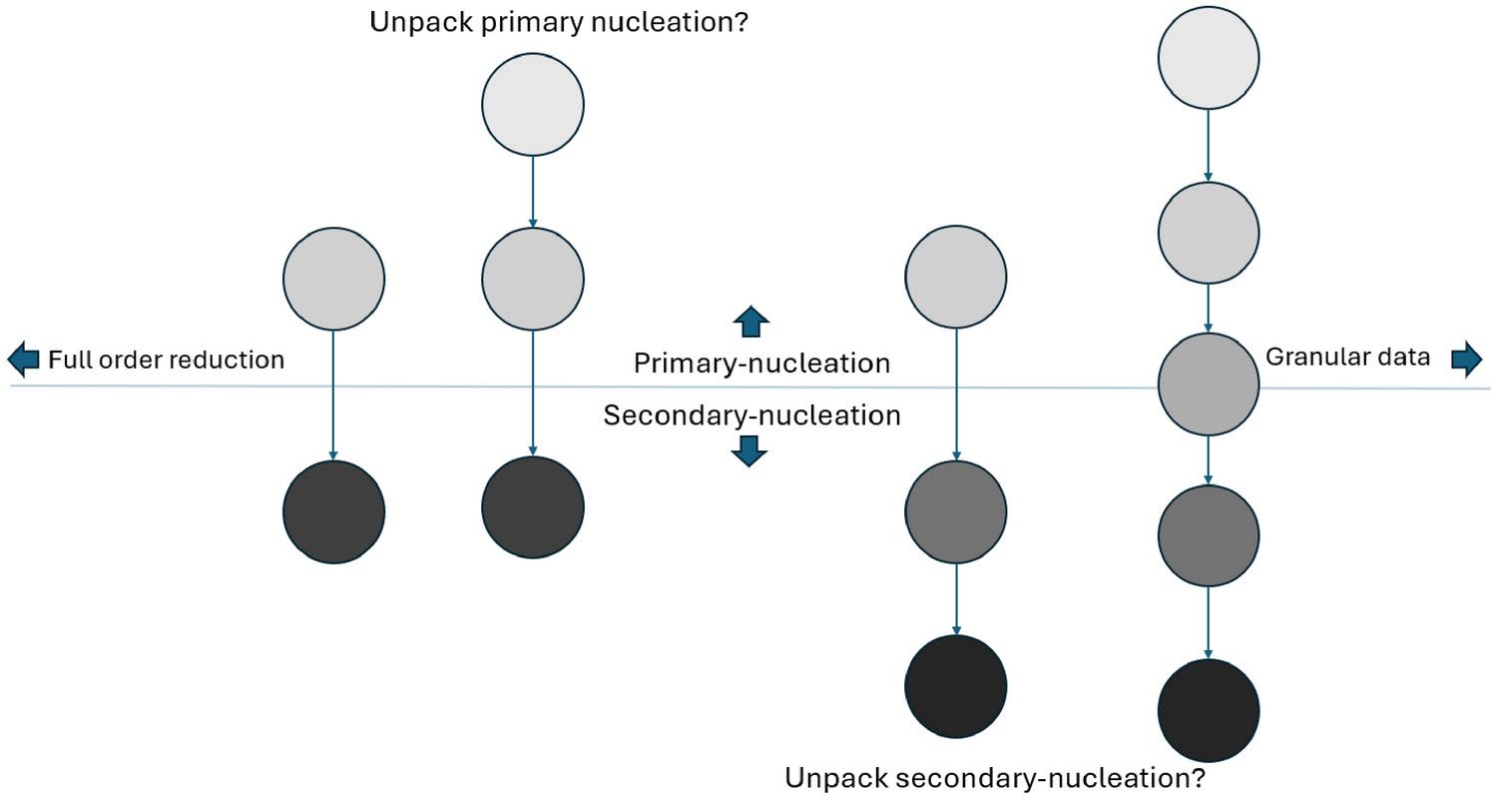
A diagram of the on-pathway model reduction choice confronting the problem of optimal reduced order scaling.

Presented with a novel dataset, we propose a method for optimal model scale reduction. For example: consider a dataset with measurements of monomer, pre-nucleation oligomers, and post-nucleation fibrils. Where each measurement is marginally close enough to each neighbor to consider model dimensionality reduction in either direction. Time-series measurements in this study include two hundred uniformly discretized fibril observations of sizes; $1\mu M, 1.4\mu M, 2\mu , 3\mu M,$ and $5\mu M$. Intensity values, recorded through fibril saturation, reflect the nucleation, elongation, and saturation phases of fibril formation. Data are normalized depending upon the proposed collapse or expansion of pre- and post-nucleation reactions. The observed values at these time points are used as training targets, and the model learns to minimize the difference between its predictions and these known values. In addition to the physical data, which consists of measured values over time, the training also incorporates constraints from the governing equations. These equations ensure that the learned relationships follow the expected physical behavior of the system. The combination of observed data and equation-driven constraints allows the model to generalize beyond the given points while maintaining consistency with the underlying physical principles. Figure 3 serves as a visual guide for the question of dimensionality in this modeling problem, illustrating the possible pathways for model reduction and the decision-making process involved in selecting an optimal scale. This diagram underscores the iterative nature of the process, where each step represents a critical decision point in achieving a balance between model simplicity and the need to capture essential dynamics.

The proposed workflow for automatic reaction order reduction represents a methodological innovation that addresses these challenges. By iteratively fitting the Reduced Order Model Physics-Informed Neural Network (ROMPINN) across different scales of model reduction and assessing the fit, researchers can identify the most appropriate level of granularity for their model. This approach not only enhances the model’s reliability but also its relevance to current and future datasets. The informed reaction unpacking for automatic network model selection represents a potential significant step forward in the field, offering a systematic approach to tailoring models to the specific dynamics of amyloid aggregation. This methodological innovation not only advances our understanding of Alzheimer’s disease but also opens new avenues for the development of therapeutic interventions, illustrating the profound impact of modeling techniques on the fight against neurodegenerative diseases.

In many practical scenarios, reducing the complexity of a model without significantly compromising its accuracy is highly desirable. This process, known as model reduction, can be particularly beneficial when dealing with models that have a high complexity, defined by the number of underlying functions or governing equations.

Consider a physical system governed by two equations:

3 $$\begin{aligned} & \mathcal {L}_1(u) = f(x), \end{aligned}$$

4 $$\begin{aligned} & \mathcal {L}_2(v) = g(x), \end{aligned}$$

where $\mathcal {L}_1$ and $\mathcal {L}_2$ are differential operators, and *f*(*x*) and *g*(*x*) are known functions.

To reduce the complexity of this model, we aim to combine these two equations into a single, reduced equation. This can be achieved by finding a new function *h*(*x*) employing the laws of mass action on revised modeling agents, which combines *f*(*x*) and *g*(*x*) into a single expression that retains the essential characteristics of both. The reduced model is expressed as:

5 $$\begin{aligned} \mathcal {L}(w) = h(x), \end{aligned}$$

where $\mathcal {L}$ is a differential operator that encapsulates the combined effects of $\mathcal {L}_1$ and $\mathcal {L}_2$, and *w* is the solution of interest.

1. Fit ROMPINN for maximally reduced order model. Return residuals of fit.
2. Propose crucial primary rate-limiting steps by unpacking primary-nucleation reactions, rearrange governing reactions, and fit. Explore parameter identifiability. Backtrack without improvement.
3. Propose crucial secondary rate-limiting steps by unpacking secondary-nucleation reactions, rearrange governing reactions, and fit. Explore parameter identifiability. Backtrack without improvement.

**Fig. 4:**
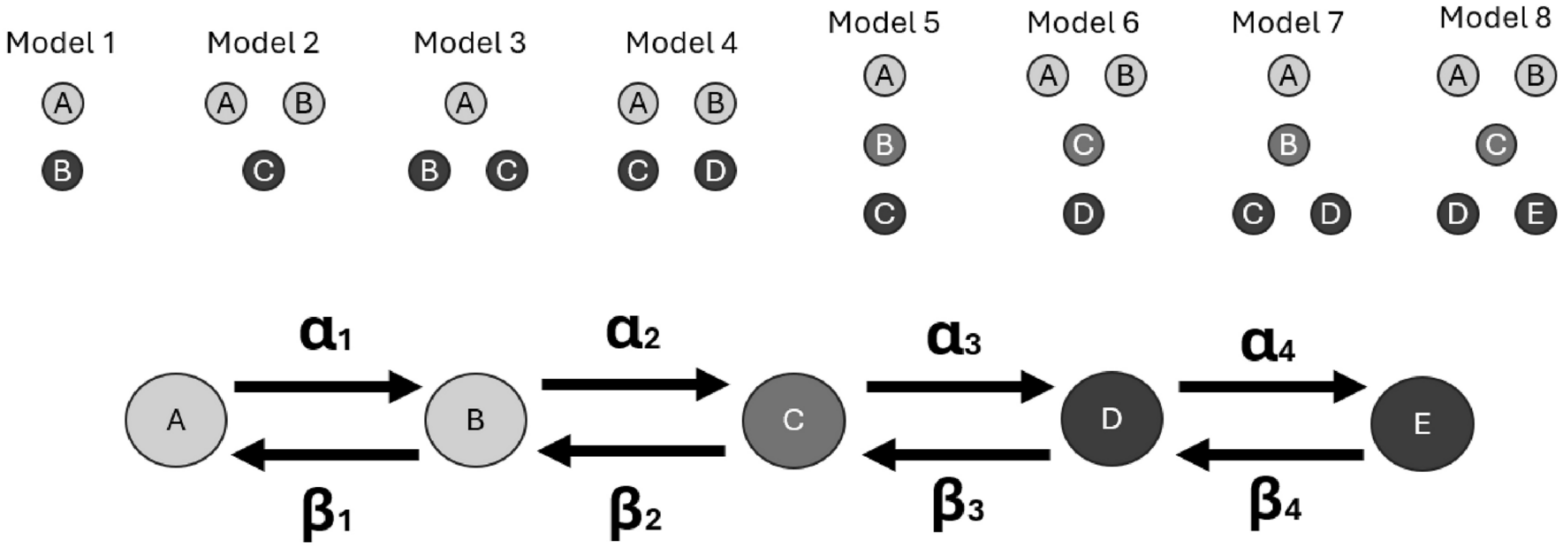
A diagram of the on-pathway model reduction choice confronting the problem of optimal reduced order scaling. Fully granular Model 8 is displayed.

Eight potential model architectures are explored in this study, each coalescing or unpacking primary and/or secondary nucleation reactions in unique ways. Figure 4 depicts the eight architectures, where Model 8 is displayed with its corresponding forward and backward reaction parameters, $\alpha$ and $\beta$ respectively.

This unpacking paradigm enables a more comprehensive examination of potential model architectures for amyloid aggregation pathways, as exemplified in Fig. 1a. By varying the selected granularity of nucleation reactions, this approach facilitates the selection of appropriate model structures for confronting new datasets. These architectures allow for the systematic application of models with tailored configurations, providing a framework that aligns with the specific dynamics of amyloid aggregation. The governing ordinary differential equations for each model, derived from the time derivatives, are presented below. Modeling variables and parameters are summarized in Tables 1 and 2.

**Model 1**

1. $$\frac{dB1\_35}{dt} = a1 \cdot c \cdot B4 - a2 \cdot c \cdot B1\_35^c$$

2. $$\frac{dB4}{dt} = a2 \cdot B1\_35^c - a1 \cdot B4$$

**Model 2**

1. $$\frac{dB1}{dt} = a1 \cdot n \cdot B1\_7 - a2 \cdot n \cdot B1^n$$

2. $$\frac{dB1\_7}{dt} = a2 \cdot B1^n - a1 \cdot B1\_7 + a3 \cdot m \cdot B4 - a4 \cdot m \cdot B1\_7^m$$

3. $$\frac{dB4}{dt} = a4 \cdot B1\_7^m - a3 \cdot B4$$

**Model 3**

1. $$\frac{dB1\_35}{dt} = a1 \cdot n \cdot B3 - a2 \cdot n \cdot B1\_35^n$$

2. $$\frac{dB3}{dt} = a2 \cdot B1\_35^n - a1 \cdot B3 + a3 \cdot m \cdot B5 - a4 \cdot m \cdot B3^m$$

3. $$\frac{dB5}{dt} = a4 \cdot B3^m - a3 \cdot B5$$

**Model 4**

1. $$\frac{dB1}{dt} = a1 \cdot n \cdot B1\_7 - a2 \cdot n \cdot B1^n$$

2. $$\frac{dB1\_7}{dt} = a2 \cdot B1^n - a1 \cdot B1\_7 + a3 \cdot m \cdot B3 - a4 \cdot m \cdot B1\_7^m$$

3. $$\frac{dB3}{dt} = a4 \cdot B1\_7^m - a3 \cdot B3 + a5 \cdot s \cdot B5 - a6 \cdot s \cdot B3^s$$

4. $$\frac{dB5}{dt} = a6 \cdot B3^s - a5 \cdot B5$$

**Model 5**

1. $$\frac{dB1\_2}{dt} = a1 \cdot n \cdot B2 - a2 \cdot n \cdot B1\_2^n$$

2. $$\frac{dB2}{dt} = a2 \cdot B1\_2^n - a1 \cdot B2 + a3 \cdot m \cdot B4 - a4 \cdot m \cdot B2^m$$

3. $$\frac{dB4}{dt} = a4 \cdot B2^m - a3 \cdot B4$$

**Model 6**

1. $$\frac{dB1}{dt} = a1 \cdot n \cdot B1\_4 - a2 \cdot n \cdot B1^n$$

2. $$\frac{dB1\_4}{dt} = a2 \cdot B1^n - a1 \cdot B1\_4 + a3 \cdot m \cdot B2 - a4 \cdot m \cdot B1\_4^m$$

3. $$\frac{dB2}{dt} = a4 \cdot B1\_4^m - a3 \cdot B2 + a5 \cdot s \cdot B4 - a6 \cdot s \cdot B2^s$$

4. $$\frac{dB4}{dt} = a6 \cdot B2^s - a5 \cdot B4$$

**Model 7**

1. $$\frac{dB1\_2}{dt} = a1 \cdot n \cdot B2 - a2 \cdot n \cdot B1\_2^n$$

2. $$\frac{dB2}{dt} = a2 \cdot B1\_2^n - a1 \cdot B2 + a3 \cdot m \cdot B3 - a4 \cdot m \cdot B2^m$$

3. $$\frac{dB3}{dt} = a4 \cdot B2^m - a3 \cdot B3 + a5 \cdot s \cdot B5 - a6 \cdot s \cdot B3^s$$

4. $$\frac{dB5}{dt} = a6 \cdot B3^s - a5 \cdot B5$$

**Model 8**

1. $$\frac{dB1}{dt} = a1 \cdot n \cdot B1\_4 - a2 \cdot n \cdot B1^n$$

2. $$\frac{dB1\_4}{dt} = a2 \cdot B1^n - a1 \cdot B1\_4 + a3 \cdot m \cdot B2 - a4 \cdot m \cdot B1\_4^m$$

3. $$\frac{dB2}{dt} = a4 \cdot B1\_4^m - a3 \cdot B2 + a5 \cdot s \cdot B3 - a6 \cdot s \cdot B2^s$$

4. $$\frac{dB3}{dt} = a6 \cdot B2^s - a5 \cdot B3 + a7 \cdot p \cdot B5 - a8 \cdot p \cdot B3^p$$

5. $$\frac{dB5}{dt} = a8 \cdot B3^p - a7 \cdot B5$$

**Table 1:** Definition of the variable in Models 1 through 8.

Variable	Definition
$B1$	Concentration of the first species involved in the reaction.
$B1\_2$	Intermediate species related to $B1$ and a higher-order aggregate.
$B1\_4$	The second species involved in the reaction.
$B1\_7$	Another intermediate species derived from $B1$ and a higher-order aggregate.
$B1\_35$	Another intermediate species derived from $B1$ and a higher-order aggregate.
$B2$	The third species involved in the reaction.
$B3$	The fourth species involved in the reaction.
$B4$	Another intermediate species derived from $B3$ and $B5$ aggregate.
$B5$	The fifth species involved in the reaction.

**Table 2:** Definition of the parameters in Models 1 through 8.

Parameter	Definition
$a1$	Reaction rate constant for the formation or transformation of species $B1$.
$a2$	Reaction rate constant for the consumption or transformation of species $B1$.
$a3$	Reaction rate constant for the formation or transformation of species $B2$.
$a4$	Reaction rate constant for the consumption or transformation of species $B2$.
$a5$	Reaction rate constant for the formation or transformation of species $B3$.
$a6$	Reaction rate constant for the consumption or transformation of species $B3$.
$a7$	Reaction rate constant for the formation or transformation of species $B5$.
$a8$	Reaction rate constant for the consumption or transformation of species $B5$.
$n,m,s,c,p,c$	Exponent or order of the reaction for various species interactions.

This model reduction approach explores the model architecture space in a limited scope—adding or removing granularity for primary or secondary nucleation species. Future work must undoubtedly include exploration of other important rate limiting factors toward pathogenesis. The aggregation process encompasses several biophysical processes that contribute to the formation and accumulation of A$\beta$ fibrils, which are central to the disease. Several other models are explored employing Amylofit, and further reinforce the need to include other subject area expertise in the generic model reduction architecture. We briefly summarize these models in the following.

First, nucleation and elongation model posits that A$\beta$ aggregation begins with a nucleation phase, where monomers come together to form a stable nucleus, followed by an elongation phase, where additional monomers add to the growing fibril. The nucleation phase is considered a rate limiting step because it requires overcoming an energetic barrier. Once a nucleus is formed, the elongation phase can proceed more rapidly. This model is important for understanding the initial stages of amyloid fibril formation and highlights the significance of targeting early nucleation events in therapeutic interventions.

In the secondary nucleation dominated model, the emphasis is on the formation of new nuclei on the surfaces of existing fibrils. This process significantly accelerates the aggregation of A$\beta$, leading to a faster accumulation of amyloid fibrils. Secondary nucleation is a key mechanism by which small changes in the concentration of A$\beta$ can lead to dramatic increases in fibril formation, underlining its importance in the exponential phase of amyloid accumulation.

The fragmentation dominated model highlights the role of fibril fragmentation in generating new seeds for further aggregation. Fragmentation increases the number of active ends, facilitating the addition of monomers and accelerating fibril formation. This process is crucial for understanding how fibril breakage contributes to the spread and severity of amyloidosis.

The fragmentation and secondary nucleation model combines aspects of both fragmentation and secondary nucleation, recognizing that both processes can significantly contribute to A$\beta$ aggregation. It suggests a synergistic effect where fragmentation creates new ends that enhance elongation, while secondary nucleation increases the number of nucleation sites. This dual mechanism provides a more comprehensive understanding of how A$\beta$ fibrils proliferate and accumulate.

Finally, the multistep secondary nucleation dominated model is considered. This this model proposes that secondary nucleation involves multiple steps, including the attachment of A$\beta$ monomers to fibril surfaces, structural changes to form a nucleus, and the eventual detachment of new nuclei to seed further fibril formation. It highlights the complexity of the nucleation process and suggests multiple potential targets for therapeutic intervention. This model underscores the intricate balance and interplay between different biophysical processes in amyloidosis and the importance of targeting specific steps in the aggregation pathway to combat diseases like Alzheimer’s.

## Results

**Fig. 5:**
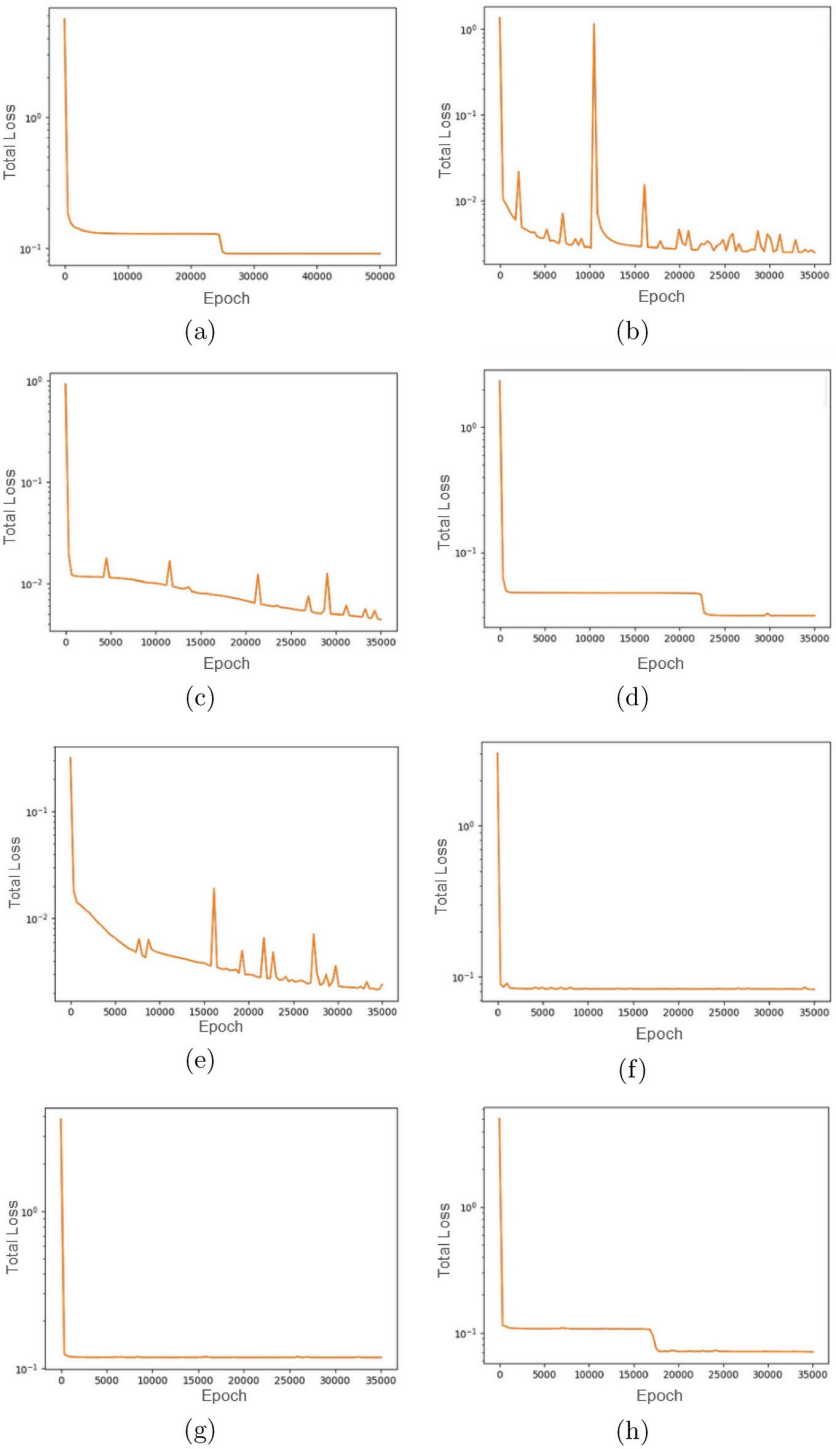
The cumulative physics-informed and observational training loss for (**a**) Model 1, (**b**) Model 2, (**c**) Model 3, (**d**) Model 4, (**e**) Model 5, (**f**) Model 6, (**g**) Model 7, and (**h**) Model 8. The y-axis is the total loss: sum of the data loss and the physics-informed loss. The x-axis shows number of epochs trained.

First, we examine the utility of ROMPINN to fit to observed data with reliable residuals. All eight reduced order binning structures are capable of fitting data with mean absolute error $< 0.08$. The model is trained with three hidden layers of size six. The activation is *tanh*; 35, 000 epochs of the *adam* optimizer with learning rate 0.001. Figure 5 displays the physics-informed and data driven cumulative training losses, while Table 3 displays the comparison between residuals fit with the ROMPINN exploratory architecture and state-of-the-art amyloid system models^5^. While mean residual values for ROMPINN are relatively consistent, the utility of models for accurate forecasting is dependent more on the binning structure and ability to fit data. The balance of fit residual, forecasting ability, and parameter identifiability is what affords a proposed model architecture *optimality* or, rather, make it the current best choice for order reduction in relation to the available observations. Identifiability for each parameter are calculated employing COPASI parameter scans^18^ and the process is detailed in Appendix B. The profile likelihood method is a statistical technique used to estimate the confidence intervals for parameters within complex models, especially when dealing with multiple parameters or constrained estimation problems. It operates by fixing the parameter of interest at various values while optimizing the likelihood function with respect to all other estimable parameters, thereby constructing a profile of the likelihood as a function of the parameter of interest. This profiling process allows for a more accurate estimation of the confidence interval for the parameter by taking into account the uncertainty and interdependence of all model parameters.

ROMPINN is generally capable at producing reliable fits to the data, as displayed in Table 3 and Fig. 5, while showcasing the dual purpose of the PINN module for reaction parameter estimation in Table 5. ROMPINN generally produces tighter fit to observed data than traditional least squares methods. State-of-the-art methods particularly fine-tuned for A$\beta$ aggregation modeling outperform in terms of fit to transient concentrations^5^. However the generalizability of the ordered aggregation model with respect to off-pathway aggregation (simply by adding simulation catalyst *L* as in Fig. 1), coupled with reliably identifiable parameters, allow for further lines of inquiry for seeding studies,and stability of various off-pathways toward particularly toxic pathogenic states^16^. Each iteration of the PINN itself is susceptible to overfitting of the particular dataset, in the context of parameter estimation using a PINN, overfitting to a specific dataset is expected following that the primary objective is to fine-tune the model parameters to accurately reflect the dynamics within that dataset. Unlike traditional machine learning tasks where generalization to unseen data is crucial, parameter estimation relies on the PINN capturing the detailed features of the dataset at hand. The PINN retains the ability to better align with the observed data while respecting the physical constraints embedded in the model, allowing for precise parameter values.

The incorporation of physical laws within the PINN helps mitigate the typical risks of overfitting by ensuring the model adheres to plausible and interpretable solutions. This means that even when the model closely fits the data, it remains grounded in established physics principles. Furthermore, since parameter estimation is typically performed iteratively, overfitting in each iteration supports incremental refinement, where the model can adjust parameters more accurately based on the dataset’s nuances. Ultimately, the trade-off of overfitting is justified by the need for accurate parameter values that reflect the specific system being studied, making it a valuable approach for this particular task.

**Fig. 6:**
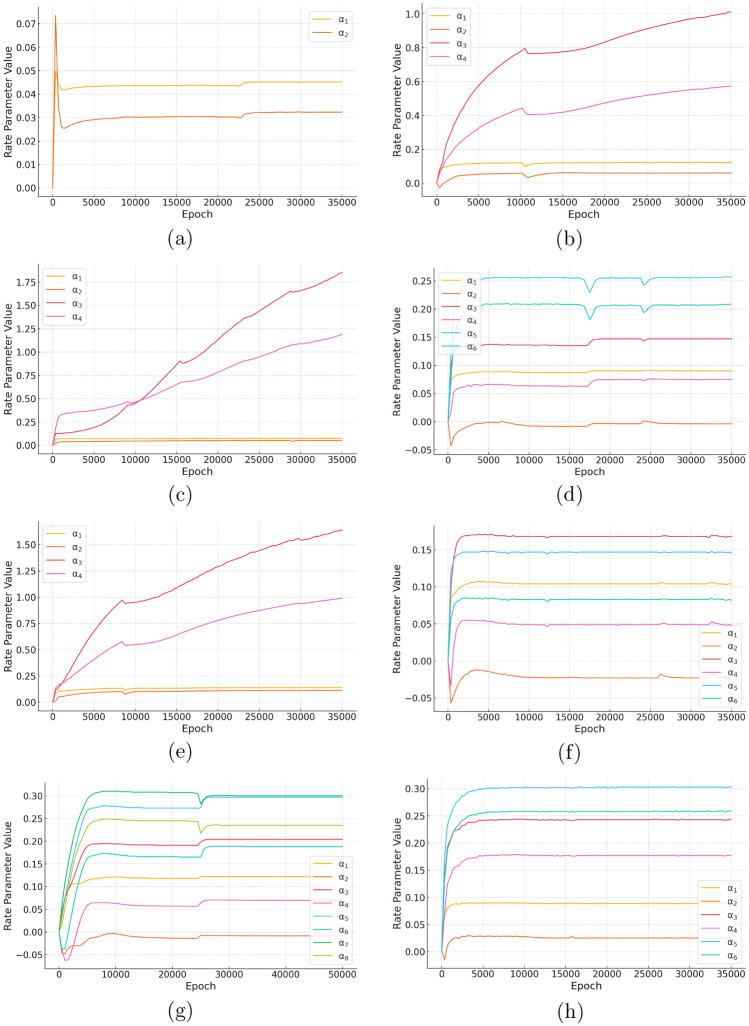
The learned rate parameters for (**a**) Model 1, (**b**) Model 2, (**c**) Model 3, (**d**) Model 4, (**e**) Model 5, (**f**) Model 6, (**g**) Model 7, and (**h**) Model 8. The y-axis displays the parameter value as it varies with the x-axis: the number of epochs trained.

Figure 6 illustrates the evolution of reaction rate parameters over training epochs, showcasing their convergence and stability in response to physics-informed loss minimization. By enforcing physical constraints during learning, ROMPINN enables the estimation of parameters that are not only statistically optimal but also physically meaningful. The incorporation of physics-informed loss ensures that the optimization process does not merely overfit to the observed data but remains aligned with underlying biophysical principles. This study further underscores the role of secondary nucleation and fragmentation as critical processes in amyloid aggregation.

**Fig. 7:**
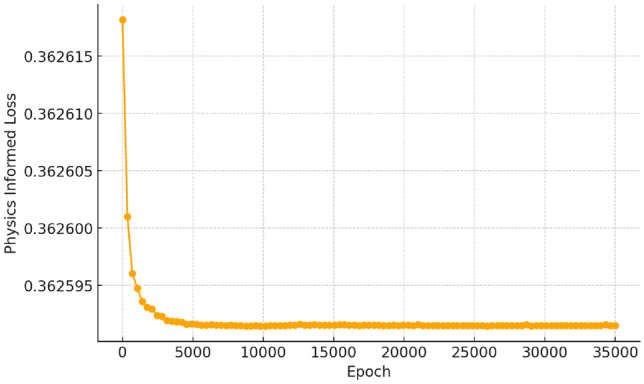
The physics-informed portion of the loss for Model 7 as a function of training epoch.

The learned rate parameter evolution in Fig. 6 highlights differences in stability, identifiability, and complexity across reduced-order models. Minimalist models, such as Models 1 and 6 (Fig. 6a,f), converge quickly and remain stable but oversimplify aggregation dynamics, failing to fully capture secondary interactions. In contrast, moderate complexity models (Models 3, 5, and 7; Fig. 6c,e,g) offer a balance between capturing key reaction processes and maintaining computational feasibility. Model 7 stands out as the most stable and well-constrained, reinforcing the dominant role of secondary nucleation while avoiding excessive parameter sensitivity. The physics-informed loss associated with the learned parameters for Model 7 are shown in Fig. 7. The proportionality between physics-informed loss and learned rate parameters reflects the stability and identifiability of reduced-order models. In well-constrained models like Model 7, a lower and smoothly decreasing physics-informed loss corresponds to stable parameter evolution. This relationship underscores the necessity of balanced complexity in model selection to ensure both physical fidelity and numerical stability

High-granularity models, including Models 2, 4, and 8 (Fig. 6b,d,h), introduce instability due to over-parameterization. Model 8, in particular, exhibits frequent oscillations, indicating overfitting and sensitivity to redundant reaction pathways. These results emphasize that moderate complexity models, particularly Model 7, provide the best balance of stability and interpretability. While minimalist models lack mechanistic depth and high-complexity models suffer from instability, ROMPINN’s systematic model reduction helps refine reaction networks while preserving physical meaning. This study reinforces the need for structured model selection to balance complexity, stability, and generalizability in reduced-order modeling. Navigating these tradeoffs between minimalist and high-complexity models, and the competing affects on learning machine optimization routines is a hallmark challenge of physics-informed machine learning.

**Table 3:** Comparison between ROMPINN and state-of-the-art amyloid fitting tools.

Levenberg-Marquardt	
Model 8	0.10912
ROMPINN	
Model 1	0.054556
Model 2	0.016991
Model 3	0.021339
Model 4	0.055874
Model 5	0.020794
Model 6	0.075503
Model 7	0.080127
Model 8	0.060492
Amylofit	
Nucleation, elongation	0.0703
Secondary nucleation dominated	0.001019
Fragmentation dominated	0.000489
Fragmentation and secondary nucleation	0.000913
Multistep secondary nucleation dominated	0.057

Additionally, the robustness of the ROMPINN framework with respect to timescale granularity is evaluated and compared to Amylofit models. Table 4 presents the mean squared error of fit for two Amylofit models and eight ROMPINN models. Notably, the Amylofit models do not exhibit performance improvement as the volume of available data increases. In contrast, the fitness of the ROMPINN models scales with the available data. Conclusively, this demonstrates the ROMPINN framework’s ability to maintain data fits and extract physically meaningful rate parameters, while leveraging a key advantage of machine learning: scalability and adaptability to larger datasets. Error values have been normalized by the maximum separately for each method to more readily track the change in model fitness as timescale varies.

**Table 4:** Mean squared error of fit for two Amylofit models and eight ROMPINN models at different timescales (values rounded to the hundred-thousandth place).

	Amylofit Multi.	Amylofit Nucl., Elon.	Model 8	Model 7	Model 6	Model 5	Model 4	Model 3	Model 2	Model 1
50	0.64205	0.92437	0.10042	0.99927	0.99914	0.99956	1.00000	1.00000	1.00000	1.00000
60	0.82670	0.92857	0.09979	0.98001	0.99533	1.00000	0.99891	0.99470	0.99742	0.99734
70	0.78409	0.91597	0.09973	1.00000	0.99640	0.99303	0.99128	0.99210	0.99182	0.99094
80	0.65625	0.91176	0.10103	0.97045	0.98665	0.99092	0.98503	0.98204	0.98581	0.98104
90	0.65341	0.92857	0.09836	0.96791	0.98644	0.98492	0.97769	0.97600	0.98292	0.97991
100	0.66477	0.89076	1.00000	0.96218	1.00000	0.97268	0.96150	0.95981	0.96915	0.96320
125	1.00000	1.00000	0.97827	0.96192	0.98113	0.97873	0.97105	0.96946	0.97936	0.97053
150	0.65909	0.90756	0.97236	0.95357	0.99285	0.97283	0.96279	0.95849	0.96879	0.96137
175	0.67045	0.92437	0.99412	0.95920	0.99579	0.97375	0.96140	0.96026	0.96954	0.96351
200	0.66477	0.92017	0.09614	0.94676	0.97300	0.95989	0.94511	0.94038	0.95435	0.94207

Column one indicates how many time points are sampled for each experiment.

Table 5 details the estimated parameters from each proposed model architecture. Note when binning is done fully, only one reversible reaction remains: where $\alpha$ and $\beta$ represents forward and backward rates respectively. However, the fully unpacked model contains eight parameters to be estimated.

**Table 5:** Reaction rate parameters estimated by ROMPINN routine.

	$\alpha _1$	$\alpha _2$	$\alpha _3$	$\alpha _4$	$\beta _1$	$\beta _2$	$\beta _3$	$\beta _4$
Model 1	0.0452	X	X	X	0.0323	X	X	X
Model 2	0.122	1.01	X	X	0.0604	0.573	X	X
Model 3	0.0747	1.85	X	X	0.0516	1.19	X	X
Model 4	0.0902	0.257	0.147	X	− 0.00389	0.751	0.209	X
Model 5	0.141	1.64	X	X	0.114	0.0991	X	X
Model 6	0.104	0.146	0.168	X	− 0.0228	0.485	0.0821	X
Model 7	0.0888	0.0243	0.303	X	0.0254	0.177	0.259	X
Model 8	0.122	0.204	0.297	0.3	− 0.00858	0.0697	0.188	0.23

**Fig. 8:**
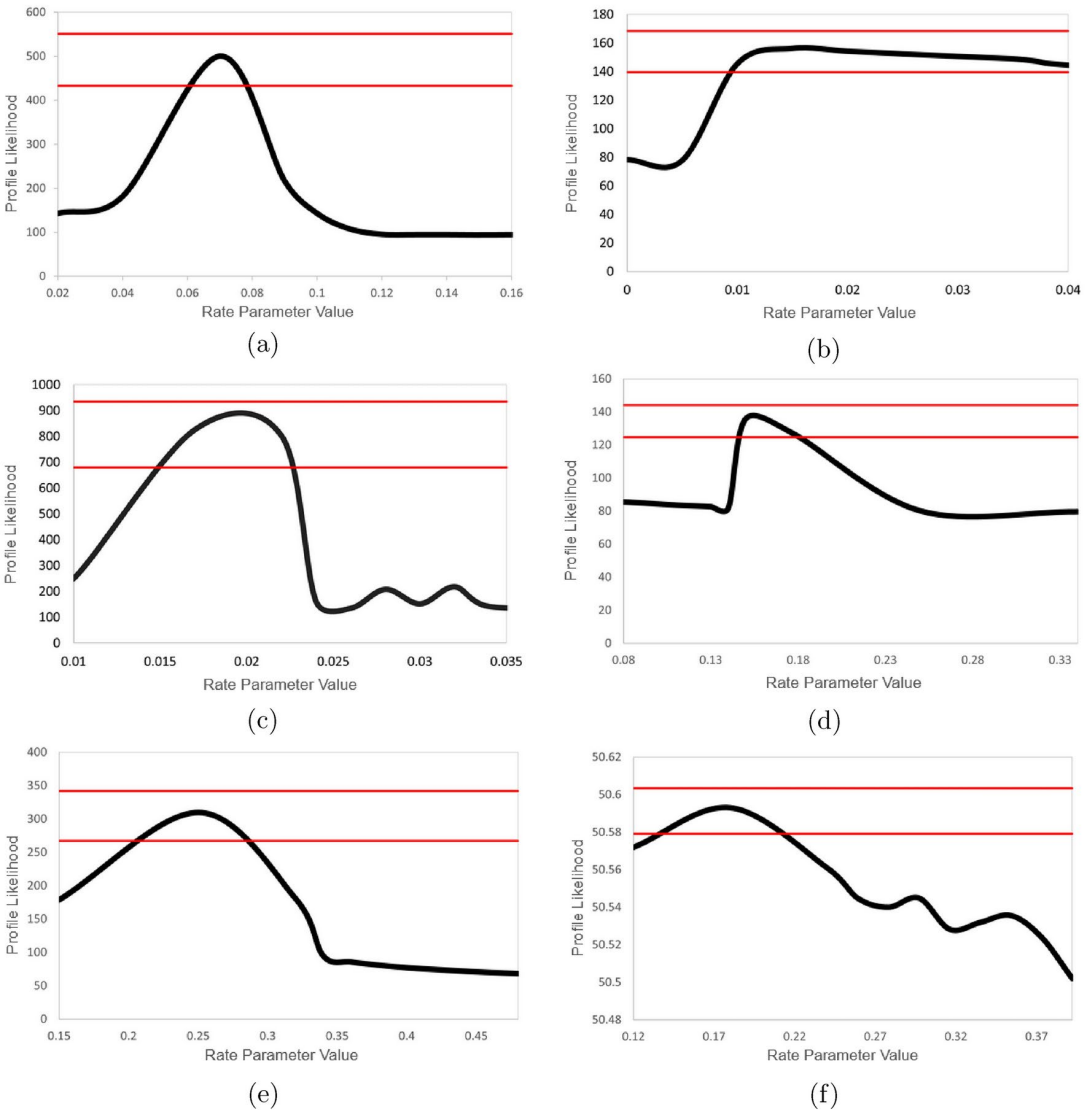
Likelihood profile for parameters estimated in Model 7. The y-axis displays the profile likelihood as a function of the varied estimable parameters on the x-axis $\alpha _i$ (**a**, **c**, **e**) and $\beta _i$ (**b**, **d**, **f**) where i = 1, 2, and 3. $95\%$ confidence intervals displayed about the maximum.

Generally, in employing parameter scans of the ordered aggregation network with Copasi parameter scans^18^, the parameters of Model 7 are mostly robust toward large permutations of the conditions. Likelihood profiles in Fig. 8 reflect generally acceptable symmetry about the maximum likelihood estimate in reaction rates under scan: suggesting those rates are significantly determining steps in the reaction network. The task of comparing model architecture, PINN fit to data, and parameter identifiability suggest Model 7 the most apt—agreeing with the well understood importance of secondary nucleation reactions, while nodding toward the possibility of other important post-nucleation steps like fragmentation. Profile likelihoods with tight $95\%$ confidence intervals about the maximum likelihood estimates suggest individually identifiable parameters. The relatively better identifiabilty in backwards, secondary nucleation reaction steps further suggests an important underlying rate-limiting process: further providing concordance with the understanding about the importance of secondary nucleation and fragmentation interactions.

3

## Conclusions

The integration of machine learning, and particularly physics-informed neural networks, into the study of A$\beta$ aggregation represents a paradigm shift in how we approach the modeling of complex biochemical processes. As the data landscape in amyloid research continues to evolve, with increasingly sophisticated observational techniques generating ever-larger datasets, the demand for models that can dynamically adapt and scale to new information becomes more pressing. The methodology presented in this paper offers a flexible, scalable solution to this challenge, insofar as the ROMPINN parameter estimation and identifiability analysis enable the study of model architectures on various scales, and the guided exploration of potential architectures.

In general, no such automatic model reduction technique currently exists in the literature. The current work uses PINN for parameter estimation, so we enumerate possible model architectures and find the best fit through PINN. The parameter estimation block tries to estimate the model parameters that fits all of the experimental time-series data, so this is essentially a global fitting method. The specific advantage in this PINN technique is that it tends to get better with more time-series data while we should see the opposite trend with established methods.

The development of automatic network models for A$\beta$ aggregation is urgent for several reasons. First, an avenue toward studying the complexity vs. reliability trade-off: as the complexity of the biological system increases, so does the challenge of capturing all relevant dynamics without oversimplifying the system. Automatic network models offer a pathway to navigate this trade-off, enabling the selection of a modeling scale that retains essential features while remaining computationally feasible. Understanding the detailed mechanisms of A$\beta$ aggregation is essential for developing effective treatment strategies for Alzheimer’s disease. Automatic network models can accelerate this process by providing insights into how changes at the molecular level influence the formation of toxic fibrils. Finally, the PIML framework enables adaptability to new data through reliable simulation and parameter estimation: the field of amyloid research is rapidly evolving, with new data constantly emerging. Automatic models that can adapt to new datasets and refine their parameters accordingly are crucial for keeping pace with advancements in the field.

Moreover, the implications of this research extend beyond Alzheimer’s disease. The approach outlined here is applicable to a wide range of biochemical and biophysical systems characterized by complex, multiphysics interactions. Exploring the dimensionality of network models in such applications is a crucial first step toward modeling and simulation. By providing a framework for automatic model reduction that balances the competing demands of computational efficiency and scientific accuracy, this work paves the way for more effective and targeted therapeutic strategies across various domains. Future work will explore the generalizability of this framework to other types of protein aggregation, as well as its potential applications in the broader field of systems biology. This work is enabled by the usefulness of physically relevant prior knowledge about ordered aggregation, our future work intends to apply the PINN framework for exploring reduced model architectures to determine: key components in pharmacodynamic models of immune response in COVID infection^21^; critical pathways in the computational macrophage polarization system^22^.

The continued refinement of PINN architectures and routines for improving the optimization of data loss and physics-informed loss both jointly and independently, combined with advances in experimental techniques, promises to unlock new insights into the fundamental mechanisms of disease and open new avenues for intervention.

## Supplementary Information

Supplementary Information.

## Data Availability

All data and code used in the analyses presented in this manuscript are openly accessible on GitHub at https://github.com/Bluewaves54/abetaPINN. This repository contains the original datasets, scripts, and instructions necessary to reproduce the results and analyses detailed in this work.
