# Supplementary material for: Physics-informed machine learning for automatic model reduction in chemical reaction networks
Source: Sci Rep. 2025 Mar 7;15:7980. doi: 10.1038/s41598-025-92680-8 (PMC11889170; doi:10.1038/s41598-025-92680-8)
Supplement: Supplementary file 1 — Supplementary Information. [file 41598_2025_92680_MOESM1_ESM.pdf]

A Model Derivation

Each arrow in our network abstraction (presented in Figure ??) corresponds to a coalescing or dissolving aggregation reaction. These reactions follow the law of mass action, allowing us to transform the list of chemical reactions into a system of differential equations that describe the concentration of each Amyloid-β species. The governing equations reflect the dynamics of aggregation, dissociation, and conversion of the various oligomers in the system. The parameters associated with these reactions are summarized in Table ??.

The system of equations that governs the evolution of the concentrations of each Amyloid-β species is given by:

$$
\begin{aligned}
\frac{dA_1}{dt} &= nk_2^- A_n - nk_2^+ A_1^n + k_1^- A_1' - k_1^+ A_1 L, \\
\frac{dA_1'}{dt} &= nk_3^- A_n' - nk_3^+ A_1'^n + k_1^+ A_1 L - k_1^- A_1', \\
\frac{dA_n}{dt} &= k_2^+ A_1^n + \frac{m}{n} k_5^- A_m + k_4^- A_n' - k_2^- A_n \\
&-k_4^+ A_n L^n - \frac{m}{n} k_5^+ A_n^{\frac{m}{n}}, \\
\frac{dA_n'}{dt} &= k_3^+ A_1'^n + k_4^+ A_n L^n + \frac{m}{n} k_6^- A_m' - k_3^- A_n' \\
&-\frac{m}{n} k_6^+ A_n' - k_4^- A_n^{\frac{m}{n}}, \\
\frac{dA_m}{dt} &= k_5^+ A_n^{\frac{m}{n}} - k_5^- A_m, \\
\frac{dA_m'}{dt} &= k_6^+ A_n'^{\frac{m}{n}} - k_6^- A_m,
\end{aligned}
$$

The reaction constants are defined as:

$$
\begin{aligned}
\alpha_1 &= \frac{k_2^-}{k_1^-}; \alpha_2 = \frac{k_2^+ A_0^{n-1}}{k_1^-}; \alpha_3 = \frac{k_1^+ L}{k_1^-}; \alpha_4 = \frac{k_4^+ L}{k_1^-}; \\
\alpha_5 &= \frac{k_5^-}{k_1^-}; \beta_1 = \frac{k_3^-}{k_1^-}; \beta_2 = \frac{k_3^+ A_0^{n-1}}{k_1^-}; \beta_3 = \frac{k_5^+ A_0^{\frac{m}{n}-1}}{k_1^-}; \\
\beta_4 &= \frac{k_4^-}{k_1^-}; \beta_5 = \frac{k_6^-}{k_1^-}; \beta_6 = \frac{k_6^+ A_0^{\frac{m}{n}-1}}{k_1^-}
\end{aligned}
$$

This system is then non-dimensionalized. Using A_0 as the characteristic concentration of monomers and $\frac{1}{k_1^-}$ as the characteristic time, we define the dimensionless species as follows:

$$B_1 = \frac{A_1}{A_0}; B_n = \frac{A_n}{A_0}; B_m = \frac{A_m}{A_0}; \tag{1}$$

$$B_1^{'} = \frac{A_1^{'}}{A_0}; B_n^{'} = \frac{A_n^{'}}{A_0}; B_m^{'} = \frac{A_m^{'}}{A_0} \tag{2}$$

B Parameter Identifiability

Profile likelihood is a statistical method particularly useful for evaluating the confidence intervals of parameters in complex models. The likelihood function for a set of parameters θ (of interest) and ψ (nuisance parameters) based on observed data X is given by:

$$L(\theta, \psi; X)$$

The profile likelihood for θ is defined by maximizing the likelihood function over the nuisance parameters ψ for each fixed value of θ:

$$L_P(\theta; X) = \max_{\psi} L(\theta, \psi; X)$$

To estimate a confidence interval for θ, we use the profile likelihood ratio:

$$R(\theta) = \frac{L_P(\theta; X)}{L_P(\hat{\theta}; X)}$$

where $\hat{\theta}$ is the maximum likelihood estimate of θ, obtained by maximizing $L_P(\theta; X)$ over θ.

A 95% confidence interval for θ can then be approximated by finding the values of θ for which $R(\theta)$ exceeds a critical value, typically based on the chi-square distribution:

$$\{\theta : -2 \log R(\theta) \leq \chi^2_{1,0.05}\}$$

This method adjusts the likelihood function to account for the uncertainty in the nuisance parameters, providing a more accurate and robust estimation of the confidence interval for the parameter of interest.
